# Unconstrained and Contactless Hand Geometry Biometrics

**DOI:** 10.3390/s111110143

**Published:** 2011-10-25

**Authors:** Alberto de-Santos-Sierra, Carmen Sánchez-Ávila, Gonzalo Bailador del Pozo, Javier Guerra-Casanova

**Affiliations:** Group of Biometrics, Biosignals and Security, Universidad Politécnica de Madrid, Campus de Montegancedo s/n, 28223 Pozuelo de Alarcón, Madrid, Spain; E-Mails: csa@cedint.upm.es (C.S.-A.); gbailador@cedint.upm.es (G.B.P.); jguerra@cedint.upm.es (J.G.-C.)

**Keywords:** contactless hand biometrics, invariant feature extraction, security, pattern recognition, image processing, hand geometry, unconstrained biometrics

## Abstract

This paper presents a hand biometric system for contact-less, platform-free scenarios, proposing innovative methods in feature extraction, template creation and template matching. The evaluation of the proposed method considers both the use of three contact-less publicly available hand databases, and the comparison of the performance to two competitive pattern recognition techniques existing in literature: namely Support Vector Machines (SVM) and *k*-Nearest Neighbour (*k*-NN). Results highlight the fact that the proposed method outcomes existing approaches in literature in terms of computational cost, accuracy in human identification, number of extracted features and number of samples for template creation. The proposed method is a suitable solution for human identification in contact-less scenarios based on hand biometrics, providing a feasible solution to devices with limited hardware requirements like mobile devices.

## 1. Introduction

At present, trends in biometrics are inclined to provided human identification and verification without requiring any contact with acquisition devices. The point of aiming contact-less approaches for biometrics regards the upward concerns with hygiene and final user acceptability.

Concretely, hand biometrics usually have made use of a flat platform to place the hand, facilitating not only the acquisition procedure but also the segmentation and posterior feature extraction. Consequently, hand biometrics is evolving to contact-less, platform-free scenarios where hand images are acquired in free air, increasing the user acceptability and usability.

However, this fact provokes an additional effort in segmentation, feature extraction, template creation and template matching, since these scenarios imply more variation in terms of distance to camera, hand rotation, hand pose and unconstrained environmental conditions. In other words, the biometric system must be invariant to all these former changes.

The presented method proposes a hand geometry biometric system oriented to contact-less scenarios. The main contribution of this paper is threefold: firstly, a feature extraction method is proposed, providing invariant hand measurements to previous changes; second contribution consists of providing a template creation based on hand geometric distances, requiring information from only one individual, without considering data from the rest of individuals within the database; finally, a proposal for template matching is proposed, minimizing the intra-class similarity and maximizing the inter-class likeliness.

The proposed method is evaluated using three publicly available contact-less, platform-free databases. In addition, the results obtained with these databases will be compared to the results provided by two competitive pattern recognition techniques, namely Support Vector Machines (SVM) and *k*-Nearest Neighbour, often employed within the literature.

Finally, the layout of this paper remains as follows: First of all, a literature review is carried out in Section 2. Secondly, the feature extraction method is described in Section 3.2, together with a description of the database involved in the evaluation (Section 4). Afterwards, the comparative evaluation and the corresponding results are presented in Section 5. Finally, conclusions and future work are introduced in Section 6.

## 2. Literature Review

Hand biometric systems have evolved from early approaches which considered flat-surface and pegs to guide the placement of the user’s hand [1–3], to completely platform-free, non-contact techniques were user collaboration is almost not required [4–7]. This development can be classified into three categories according to the image acquisition criteria [8]:

- Constrained and contact based. Systems requiring a flat platform and pegs or pins to restrict hand degree of freedom [2,3].
- Unconstrained and contact based. Peg-free scenarios, although still requiring a platform to place the hand, like a scanner [6,9].
- Unconstrained and contact-free. Platform-free and contact-less scenarios where neither pegs nor platform are required for hand image acquisition [5,10].

In fact, at present, contact-less hand biometrics approaches are increasingly being considered because of their properties in user acceptability, hand distortion avoidance and hygienic concerns [11,12], and their promising capability to be extended and applied to daily devices with less requirements in terms of image quality acquisition or speed processor [9,10,13].

In addition, hand biometrics gather a wide variety of distinctive aspects and parameters to identify individuals, considering whether fingers [7,14,15], hand geometric features [2,3,6,15,16], hand contour [2,10,17], hand texture and palmprint [8,18] or some fusion of these former characteristics [7,14,19].

More specifically, geometrical features have received notorious attention and research efforts, in comparison to other hand parameters. Methods based on this strategy (like widths, angles and lengths) reduce the information given in a hand sample to a *N*-dimensional vector, proposing any metric distance for computing the similarity between two samples [20].

In opposition to this method, several schemes are proposed in literature applying different probabilistic and machine learning techniques to classify properly user hand samples. The most common techniques are *k*-Nearest Neighbours [21], Gaussian Mixture Models [3,22], naïve Bayes [21] or Support Vector Machines [9,18,21], which is certainly the most extended technique in hand biometrics due to their performance in template classification.

Nonetheless, these latter strategies present several drawbacks in comparison with distance-based approaches in terms of computational cost and efficiency, since probabilistic-based strategies require other user samples to conform an individual template. In other words, systems based on a classifier approach are trained for each of the enrolled persons, requiring samples from other enrolled individuals for a separate classification. This fact may become a computational challenge, for large-population systems [20]. However, in terms of individual identification performance, they certainly succeed in relation to current distance-based methods.

An overview on recent hand biometrics systems is presented in Table 1. This table presents the relation between the features required for identification, the method proposed, the population involved together with the results obtained, in terms of Equal Error Rate (EER).

As hand biometrics tends to contact-less scenarios, hand image pre-processing increases in difficulty and laboriousness, since less constraints are required concerning background, *i.e.*, the part behind the hand.

Several approaches in literature tackle with this problem by providing non-contact, platform-free scenarios but with constrained background, usually employing a monochromatic color, easily distinctive from hand texture [23]. More realistic environments propose a color-based segmentation, detecting hand-like pixels either based on probabilistic [16] or clustering methods [18,24]. Although, the constraints on background are less restrictive in this case, the performance of this segmentation procedure still lacks in accuracy.

However, a feasible solution for this latter scenario is based on an acquisition involving short distance to sensor. This approach considers the use of infrared illumination [9,18], due to the fact that infrared light only lighten close-to-camera regions, avoiding further regions (background) to be illuminated and therefore not acquired by the infrared camera.

Most recent trends in hand segmentation consider no constraint on background, proposing more efficient approaches based on multiscale aggregation, providing promising results in real scenarios [24]. This scenario is clearly oriented to the application of hand biometrics in mobile devices.

Moreover, hand biometrics also consider different acquisition modalities, namely 3D data acquisition [14,25], infrared cameras [9,18], scanners [6] or low-resolution acquisition devices [10,13].

Best results in Table 1 are achieved by Rahman *et al*. [26] and Kanhangad *et al*. [25]. The former work consists of applying Distance Based Nearest Neighbour (DBNN) and Graph Theory to both feature extraction and feature comparison. In contrast, the latter work presents a new approach to achieve significantly improved performance even in the presence of large hand pose variations, by estimating the orientation of the hands in 3D space and then attempting to normalize the pose of the simultaneously acquired 3D and 2D hand images.

As a conclusion, contact-less hand biometrics is receiving an increasing attention in recent years, and many aspects remain unresolved such as invariant feature extraction or hand template creation.

## 3. Methodology

**Figure 1.**
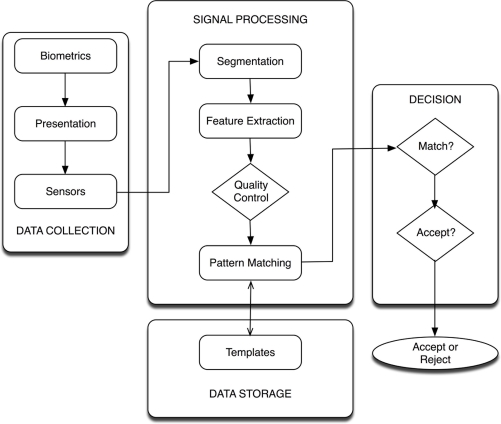
Diagram of a general biometric system.

A general biometric system involves the following steps, presented in Figure 1:

- Data Collection module is dedicated to acquired data from the biometric sensor.
- Signal Processing module involves both the pre-processing step to provide a precise segmentation and the creation of the template.
- Data Storage module stores the template, protected to ensure the biometric information is not compromised.
- Decision module provides the resolution on the identity of an individual given the template and the data collected previously.

### 3.1. Hand Image Acquisition and Pre-Processing

The contribution of this paper is focused on the Signal Processing module and Decision module, defining geometric features invariant to changes like distance to camera, hand rotation or hand pose, together with the creation of a template requiring data from one single individual instead of using data from the whole biometric database. Concerning the Decision module, this paper proposes a template matching method, which outperforms competitive pattern recognition techniques like *k*-NN and SVM (Section 5).

Contact-less biometrics impose on users almost no constraints in terms of distance to camera, hand orientation and so forth, implying a demanding pre-processing stage in terms of segmentation and contour extraction accuracy. This step is essential for a posterior precise feature extraction, and the whole hand biometric system relies strongly on this prior procedure. In addition, the proposed hand image acquisition contains no specific constraints on the characteristics of the camera

The pre-processing proposed is independent from the database, in other words, there are no specific strategies for every database. In addition, the pre-processing method contains several steps, briefly described as follows:

- Segmentation, which consists of isolating hand from background precisely.
- Finger classification, carried out after segmentation process, it consists of identifying each finger (index, middle, ring or little) correctly with independence of previous possible changes (rotation, hand orientation, pose and distance to camera).
- Valleys and tips detection, essential in order to provide accurate mark points from which features can be extracted.
- Left-Right hand classification, based on the fact that an individual can provide any hand, and the system must firstly classify the hand. Notice that without this method, fingers from left hand could be compared to fingers from right hand, resulting in errors in identification.

After introducing the main parts of the pre-processing stage, each step is explained more in detail.

Firstly, concerning segmentation, a method based on gaussian multiscale aggregation [24,29] was selected based on their properties of linearity with the number of pixels and segmentation accuracy. The proposal of this method is justified since the biometric evaluation will consider three different databases with different backgrounds and image specifications, and the multiscale aggregation strategy can provide an accurate segmentation for each database, independently on their acquisition characteristics (illumination condition, backgrounds, color or grayscale image and so forth).

This method provides a binary image as a result of the segmentation procedure, indicating which pixels correspond to hand, and which pixels to background. This binary image will be used for contour and feature extraction in Section 3.2. A deep understanding and explanation of this method is far beyond the scope of this paper.

**Figure 2.**
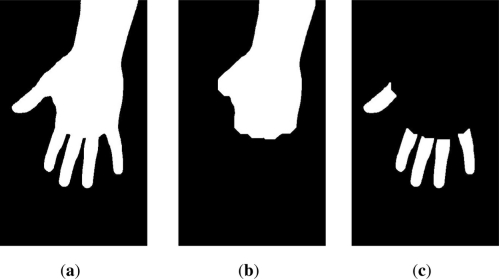
Fingers isolation steps: (**a**) represents the original segmented image, *H*; (**b**) the result after applying morphological operator (opening, disk 40), *H_p_*; (**c**) *H_f_* represents fingers after subtracting *H_p_* to *H*.

Afterwards, fingers are split from the segmented hand in order to facilitate their classification. Mathematically, let *H* be the result provided by segmentation procedure [Figure 2(**a**)]. Applying an opening morphological operator [30] with a disk structural element of size 40 will cause fingers to disappear, remaining only the part corresponding to palm. This image is named *H_p_* [Figure 2(**b**)], since it represent those pixels corresponding to palm. Although this operation is very severe, it allows conserving those region blobs which are very dense in terms of pixels, being suitable for deleting prominent blobs like fingers from hand [7].

Given *H* and *H_p_*, it is straightforward to calculate *H_f_* which represents the region blobs corresponding to fingers [five fingers, Figure 2(**c**)], by the following relation [Equation (1)]

(1) $$H_{f}=H\cdot {\bar{H}}_{p}$$

Being · an operator indicating a logical AND operation between *H* and the complementary of *H_p_*. In case, image *H_f_* contained some spurious blobs, they are erased by selecting the five most prominent blobs in image.

Figure 2 provides a visual example of the fingers isolation method.

Afterwards, five blobs are contained in *H_f_* (Figure 2) one of each corresponding to each finger. In case more than five blobs are obtained, an opening morphological operator based on a small disk structural element (size 5) will erase those small and undesired region blobs, with lack of interest for a finger classification.

In order to distinguish among fingers, all of them are classified according to two criteria: the ratio between blob length and width (eccentricity) and area (number of pixels within blob).

The blob which verifies to have the lowest values in both criteria is the little finger. The next finger with lower area is thumb, and ring, middle and index are classified according to the distance between their centroids to previous calculated fingers. In other words, that blob whose centroid is closer to little is classified as ring finger, for instance. A similar criteria was proposed by [6].

Having the finger blobs calculated, tip detection consists of calculating the finger extrema of each blob. In other words, obtain the furthest pixel in each blob in relation to a reference point. In this paper, such point coincides with the each finger centroid, due to their geometric properties of being located in the middle of each finger. Others points could be the hand centroid [10], or minimum/maximum points in contour curve [20].

Finally, since there are five fingers blobs, this method leads to five tips.

In contrast to tip detection, obtaining valleys requires more effort. Let *c* be the hand contour obtained from the edge blob in *H*. Let *t_k_* be the finger tip corresponding to finger *k*, with *k* = {*t*, *i*, *m*, *r*, *l*} meaning thumb, index, middle, ring and little respectively. In addition, *ζ_k_* = *c*(*t_k_*, *t_k_*_+1_) is the edge portion from tip *t_k_* and *t_k_*_+1_. Valley points verify to be the closest point to hand centroid *h_c_*. However, only little-ring, ring-middle and middle-index valleys support this criterion. The valley corresponding to index-thumb will be treated separately.

Then, the former valleys are calculated according to Equation (2)

(2) $$v_{k}=\text{arg}\;\underset{k}{\text{min}}(||\zeta_{k}-h_{c}||)$$

Notice that valley detection is a considerable challenging task, given that some fingers could be together one to each other, making difficult the valley point calculation.

Finally, last step consists of classifying the hand as left or right for a proper posterior feature comparison, with the aim of avoiding features from the same finger but from different hands.

Thus, hand can be classified as right or left by using three points: *t_t_*, *t_l_* and *h_c_*. Two vectors are considered, joining *h_c_* to each point tip *t_t_* and *t_l_*, which are represented by *v_T_* and *v_L_* respectively. These former vectors are on the same plane, so that their cross-vector product will be normal to that plane.

There exist a direct relation between right-left hand classification and vector *v_T_* × *v_L_*. The sign of the *z* component of *v_T_* × *v_L_* is associated with right hand, in case the sign is positive and left hand, otherwise.

In addition, this image pre-processing achieved second position in the Hand Geometric Points Detection International Competition HGC2011 [31].

### 3.2. Feature Extraction

The proposed method extracts features by dividing the finger from the basis to the tip in *m* parts. Each of these former parts measures the width of fingers, based on the Euclidean distance between two pixels. Afterwards, for each finger, the *m* components are reduced to *n* elements, with *n < m*, so that each *n* component contains the average of $\left\lfloor \frac{m}{n}\right\rfloor$ values, gathering mean value, *μ* and standard deviation *σ*. In other words, template is extracted based on an average of a finger measures set, being more reliable and precise than one single measure. This approach provides a novelty if compared to previous works in literature, where more simple measures were considered [2,3,21].

Thus, the template can be mathematically described as follows. Let *F* = {*f_i_*, *f_m_*, *f_r_*, *f_l_*} be the set of possible fingers, namely index, middle, ring and little, respectively.

Each finger *f_k_* is divided into *m* parts from basis to top, resulting in the set of widths Ω_*f*_*k*__ = {*ω*_1_, …, *ω_m_*}. From set Ω, the template is represented by $\Delta_{f_{k}}=\frac{1}{\delta_{f_{k}}}\left\{\delta_{1}^{f_{k}},\ldots ,\delta_{n}^{f_{k}}\right\}$, where each $\delta_{t}^{f_{k}}$ is defined as the average value of at least $\left\lfloor \frac{m}{n}\right\rfloor$ components in Ω_*f*_*k*__. Notice that this division could imply that last element *δ_n_* could be the average of more than $\left\lfloor \frac{m}{n}\right\rfloor$ components in order to ensure that every element in Ω_*f*_*k*__ is considered to create Δ_*f*_*k*__. In addition, *δ̄*_*f*_*k*__ represent the width arithmetic average, providing the normalization for vector Δ_*f*_*k*__.

Therefore, each hand sample is represented by a *M* = 4 × *n* components vector Δ = {Δ_*f*_*k*__} with *k* ∈ {*i*, *m*, *r*, *l*}, where the initials stand for index, middle, ring and little finger. Thumb is not considered due to its great variability in terms of movement, flexibility and orientation [18].

The width average normalization proposed for each Δ_*f*_*k*__ attempts to provide independence on several acquisition changes like hand rotation, distance to camera and invariance to small differences in pose. In contrast to the normalization provided in the literature based on finger length [3,18,20], a normalization oriented to average width contains the same properties in terms of invariability against distance to camera and rotation, but with the benefit of providing also independence on pose position respect to camera.

In order to evaluate the performance of both normalization strategies, four scenarios are proposed with different changes in acquisition. First, features are extracted from samples in natural pose, as stated in Section 3.1. Second scenario considers in-plane rotation changes, within the acquisition plane. Third scenario states different separation distance between hand and camera, and finally, changes in pose orientation. These changes cover all possible degrees of freedom in hand contact-less approaches.

**Figure 3.**
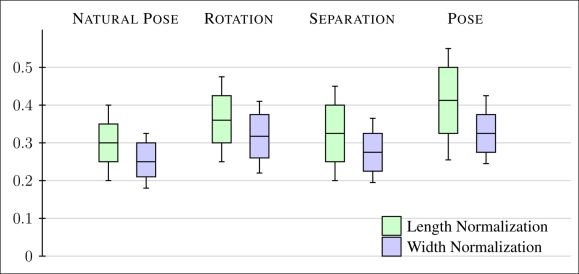
Mean and standard deviation of the difference between hand templates in different evaluation conditions (natural pose, changes in rotation, separation between hand and camera and pose orientation). The normalization based on average width provides less variation intra-class in every aspect than the finger length normalization.

Figure 3 represents the intra-class variation between features of same individuals in terms of Euclidean distance, in four different scenarios, for both normalization approaches: length (represented in green) and average width (represented in clear blue). Average value and standard deviation of the variation of extracted features in previous four scenarios are gathered, supporting the affirmation that average width normalization provides more invariant features to previous changes.

### 3.3. Template Definition

This section describes the creation of the hand template considering only samples (hand feature vectors) from a single individual, in contrast to most extended approaches in literature which propose the use of samples of all enrolled individuals on the system to create individual templates [20].

Let *W* be a *N* × *M* matrix containing *N* rows vectors of *M* components (columns) representing the *N* required samples to conform the template.

This matrix *W* is created for each individual, and it is represented by *W* = {*W*_1_, …, *W_N_*}, where each *W_i_* is a row vector containing a total of *M* components, coinciding with the number of distances contained in each extracted vector from a hand acquisition.

Let *W̃* be a $\left(\begin{array}{c} N\\ 2 \end{array}\right)\;\times \;M$ matrix, representing the absolute Euclidean difference between every pair of row vectors in *W*. In other words, *W̃* = {|*W*_1_ − *W*_2_|, |*W*_1_ − *W*_3_|, …, |*W_N−_*_1_ − *W_N_*|}, gathering a total of $\left(\begin{array}{c} N\\ 2 \end{array}\right)$ possible pairs. Matrix *W̃* represents to some extent the variation between hand acquisitions for each template position.

In fact, matrices *W* and *W̃* lead to the definition of two parameters, which are *μ^W^* and *σ^W̃^*, namely the average of extracted features and the standard deviation of the difference variation. These latter parameters attempt to collect the behaviour of all the vectors contained in *W* and the similarity between previous vectors, provided by the vector pairwise likelihood. Based on these characteristics, these vector parameters are essential to create the template.

More in detail, operators *μ* and *σ* are functions applied to matrices, defined as follows in Equations (3) and (4) respectively, ∀ *p, q* ∈ ℕ, assuming, for generalization sake, that matrix contains real values (ℝ).

(3) $$\begin{array}{l} \mu :M_{p\times q}(\mathbb{R})\to M_{1\times q}(\mathbb{R})\\ \;\;\;\;\;\;\;\;\;\;\;\;\;\;\;\;\;\;\;M\mapsto \mu^{M}={\left\{\frac{1}{p}\sum_{k=1}^{p}M_{k,j}\right\}}_{\forall j\in \{1,\ldots ,q\}} \end{array}$$

(4) $${\begin{array}{l} \sigma :M_{p\times q}(\mathbb{R})\to M_{1\times q}(\mathbb{R})\\ \;\;\;\;\;\;\;\;\;\;\;\;\;\;\;\;\;\;\;\;M\mapsto \sigma^{M}=\left\{\sqrt{\frac{1}{p}\sum_{k=1}^{p}\left(M_{k,j}-\frac{1}{q}\sum_{i=1}^{q}M_{i,j}\right)}\right\} \end{array}}_{\forall j\in \{1,\ldots ,q\}}$$

In addition, the template will consider also those *k* < *M* components which remain less invariant along different samples, *i.e.*, template will discard those components whose variability is dissimilar to some extent. This criterion is gather by vector *π*_1_*_×M_* defined as

(5) $$\pi_{i}=\{\begin{array}{ll} 1, & \text{if}\;\sigma_{i}^{\tilde{W}}\leq \mu^{\sigma^{\tilde{W}}}\\ 0, & \text{otherwise} \end{array}$$

where $\sigma_{i}^{\tilde{W}}$ corresponds to the *i*th component of vector *σ^W̃^*, and *μ*^*σ*^*W̃*^^ is the average of vector *σ^W̃^* as defined in Equation (3).

Therefore, *π* contains a “1” value in those positions where the feature variability is under the average of the variability, indicating which distances remain more invariant over acquisition.

Finally, based on this vector *π*, a last parameter is defined, which will be useful when comparing a sample (original or impostor) to a provided template. This parameter is represented by *γ*, and it is defined as the average value of the first standardized moments applied to non-null positions in *π*. In other words,

(6) $$\gamma =\frac{1}{M}\left(\frac{\mu^{\tilde{W}}}{\sigma^{\tilde{W}}}\pi^{T}\right)=\frac{1}{M}\sum_{i=1}^{M}\frac{\mu_{i}^{\tilde{W}}\pi_{i}}{\sigma_{i}^{\tilde{W}}}$$

where *π^T^* makes reference to the transposition of matrix *π*. Furthermore, parameter *γ* can be regarded as the inverse of the coefficient of variation [30], providing a dimensionless number to compare samples with widely different means.

Finally, the hand template associated to a specific user is defined as 𝒣 = (*μ^W^*, *σ^W̃^*, *π*, *γ*).

### 3.4. Matching Based on the Hand Distances Template

Provided the template 𝒣, which collects global information from samples of a same individual, it is mandatory the definition of a likelihood function able to indicate to what extent an acquire sample (impostor or genuine) is similar to previous template 𝒣.

Thus, given a hand feature vector *h*_1_*_×M_* of *M* components (as defined in Section 3.2), the likelihood function is defined as the similarity probability *p*(*h|*𝒣) given by the following relation (Equation (7)):

(7) $$p(h|𝒣)=\frac{1}{M}e^{{-\alpha HH}^{T}}$$

defining *H* as

(8) $$H=\frac{1}{\gamma }\left(\frac{h-\mu^{W}}{\sigma^{\tilde{W}}}\circ \pi \right)=\frac{1}{\gamma }\left(\sum_{i=1}^{M}\pi_{i}\frac{h_{i}-\mu_{i}^{W}}{\sigma_{i}^{\tilde{W}}}\right)$$

where operator *A* ○ *B* = [*a_ij_b_ij_*]_∀_*_i,j_* is defined as the Hadamard product, an entrywise multiplication for any two matrices *A*, *B* ∈ *M_p×q_*(ℝ), ∀ *p*, *q* ∈ ℕ. Furthermore, parameter *α* is a global value set experimentally to *α* = 0.01 for the whole biometric system.

This probability *p*(*h|*𝒣) is within the interval [0, 1], indicating that sample *h* belongs to user with template 𝒣 as *p*(*h|*𝒣) → 1, and vice versa.

Therefore, the biometric verification based on this approach can be carried out by stating a threshold *th* ∈ [0, 1], so that an individual (with template 𝒣*_k_*) accesses the system providing a sample *h_k_*, then the user is correctly verified (authenticated) if *p*(*h_k_|*𝒣*_k_*) ≥ *th*. Otherwise, the user is rejected.

Similarly, the identification is considered by considering same previous threshold *th*, so that, provided a sample of a user, *h_k_*, the system must decide whom the sample belongs to, or, whether the user is not enrolled in the system. In other words, if $\underset{i}{\text{arg}\;}(\text{max}\;p(h_{k}|{𝒣}_{i})\geq \mathrm{th})$ determines that *i* = *k* then the sample *h_k_* is properly identified, otherwise the user is not enrolled in the system.

Some approaches in literature fail in associating sample *h_k_* with a non-existing profile, since they provide the most likelihood an similar class, even if the sample provided by *h_k_* corresponds to a non-registered individual [20].

As a matter of fact, a trade-off must be achieved for *th* for the sake of an accurate performance in terms of false rejection and false acceptance [1].

This effect will be discussed under the result section (Section 5).

## 4. Databases

The proposed scheme in Sections 3.2 and 3.3 are evaluated considering three public databases.

The first database contains hand acquisitions of 120 different individuals of an age range from 16 to 60 years old, gathering males and females in similar proportion.

With the aim of a contact-less approach in hand biometrics, hand images were acquired without placing the hand on any flat surface neither requiring any removal of rings, bracelets or watches. Instead, the individual was required to open his/her hand naturally, so the mobile device (an HTC) could take a photo of the hand at 10–15 cm of distance with the palm facing the camera.

This acquisition procedure implies no severe constraints on neither illumination nor distance to mobile camera, being every acquisition carried out under natural light. In addition, it is a database with a huge variability in terms of size, skin color, orientation, hand openness and illumination conditions. In order to ensure a proper feature extraction, independently on segmentation, acquisitions were taken on a defined blue-coloured background, so that segmentation can be easily performed, focusing on hands. Both hands were taken, in a total of two sessions: During the first session, 10 acquisitions from both hands are collected; second session is carried out after 10–15 minutes, collecting again 10 images per hand. The image size provided by the device is 640 × 340 pixels. This first database is publicly available at www.gb2s.es. This database will be referred in this paper as GB2S database.

Second database is named “IIT Delhi Palmprint Image Database Version 1.0” [32], and it is a palmprint image database consisting of a hand images collection from the students and staff at IIT Delhi, New Delhi, India. This database has been acquired in the IIT Delhi campus during July 2006–Jun 2007 using a simple and touchless imaging setup. All the images are collected in the indoor environment and employ circular fluorescent illumination around the camera lens. The currently available database is from 235 users, all the images are in bitmap format. All the subjects in the database are in the age group 12–57 years. Seven images from each subject, from each of the left and right hand, are acquired in varying hand pose variations. Each of the subject is provided with live feedback to present his/her hand in the imaging region. The resolution of these images is 800 × 600 pixels. This database will be referred in this paper as IITDelhi database.

Third database acquisition setup is inherently simple and does not employ any special illumination nor does it make use of any pegs to cause any inconvenience to users. The Olympus C-3020 digital camera (1,280 × 960 pixels) was used to acquire both images from 287 individuals, with ten samples per user. The users were only requested to make sure that their fingers do not touch each other and most of their hand (back side) touches the imaging table. A further explanation of this database can be found in [33]. This database will be referred in this paper as UST database.

As a conclusion, these databases contain different acquisition procedures (population size, distance to camera, different illumination, hand rotation and the like) being a suitable evaluation frame for testing the proposed method.

## 5. Results

A complete evaluation of a biometric system must entail different aspects such as performance/identification accuracy, trade-off between false match rate and false non-match rate and dependency on the number of training samples and features. Given the variety of aspects to evaluate, this section is divided into the following parts:

- Evaluation criteria for biometric systems
- Comparative evaluation to SVM and *k*-NN employing the proposed databases
- Study of performance dependency on the number of training samples
- Study of performance dependency on the number of features
- Study of the improvement provided by the feature extraction method

In addition, temporal aspects and computational cost evaluation will be carried out within each of the previous presented sections, provided the following computer specifications: a PC computer @2.4 GHz Intel Core 2 Duo with 4GB 1067 MHz DDR3 of memory, considering that the proposed method was completely implemented in MATLAB.

### 5.1. Evaluation Criteria for Biometric Systems

There exist several types of testing for a biometric system considering a wide variety of aspects such as reliability, availability and maintainability; security, including vulnerability; conformance; safety; human factors, including user acceptance; relation between cost and benefit or privacy regulation compliance. The purpose of this section is to conduct a technical performance testing in terms of error rates. More in detail, the proposed assessment involves a technology evaluation, defined as an offline evaluation of one or more algorithms for the same biometric modality using a pre-existing or specially collected corpus of samples.

The evaluation criteria are defined by the following rates [12,34]:

- False-Non Match Rate (FNMR): Proportion of genuine attempt samples falsely declared not to match the template of the same characteristic from the same user supplying the sample.
- False Match Rate (FMR): Proportion of zero-effort impostor attempt samples falsely declared to match the compared non-self template.
- Failure-to-enroll rate (FTE): Proportion of the population for whom the system fails to complete the enrollment process.
- Failure-to-acquire (FTA): Proportion of verification or identification attempts for which the system fails to capture or locate and image or signal of sufficient quality.
- False Reject Rate (FRR): Proportion of verification transactions with truthful claims of identity that are incorrectly denied. Moreover, FRR is defined as follows: FRR = FTA + FNMR × (1 − FTA)
- False Accept Rate (FAR): Proportion of verification transactions with wrongful claims of identity that are incorrectly confirmed. Furthermore, FAR is calculated as follows: FAR = FMR × (1 − FTA)
- Equal Error Rate (EER): Rate at which both FAR and FRR coincides. In general, a system with the lowest EER is most accurate.

Table 2 contains the FTE and FTA rates for the three proposed databases: GB2S, IITDelhi and UST. These values will be taken into account in order to obtain FAR, FRR and EER rates in each evaluation scenario, as defined previously.

The behaviour of these latter parameters will be used for the evaluation across different databases, methods and dependency with variable parameters presented in Section 3.

### 5.2. Comparative Evaluation to SVM and K-NN Employing the Proposed Databases

The proposed method will be compared in terms of technical evaluation [35] to two competitive pattern recognition techniques, namely Support Vector Machines (SVM) and *k*-Nearest Neighbour (*k*-NN) [21]. Although a wide explanation of these approaches is beyond the scope of this paper, some concerns must be taken into account with reference to the manner both approaches carry out classification. Both SVM and *k*-NN create a template based on information from other individuals, in contrast to the proposed template method, where only samples from a single individual are required to conform the template.

In addition, there exist another difference concerning the similarity score provided by these methods.

As stated in previous Sections 3.2 and 3.3, the similarity score measures the similitude between a template and a collected sample. The similarity score in the SVM is considered as the distance to the corresponding hyperplane associated to the most likely class. Likewise, the similarity score in the *k*-NN is the minimum distance associated to an element within the corresponding class. In these experiments, *k* coincides with 3, providing a major voting selecting of the corresponding class, and SVM employs linear kernel functions. This SVM and *k*-NN configurations are justified since it is the most suitable value compromising both identification performance and computational cost.

Table 3 presents the Equal Error Rates obtained for each method (*k*-NN, SVM and proposed) in relation to the three employed databases in the evaluation (GB2S, IITDelhi and UST).

This table shows that SVM overcomes *k*-NN in terms of EER but the proposed algorithm improves the results obtained by both pattern recognition technique. The results obtained with the GB2S database are higher than those obtained with the other databases, since GB2S database contains more variability in terms of hand rotation, pose, distance to camera and environmental conditions (e.g., illumination).

**Figure 4.**
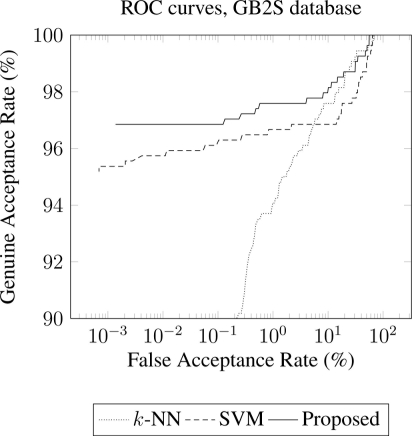
ROC curves for the proposed method in comparison to *k*-NN and SVM, using GB2S database. These results were obtained considering 4 samples for training, and 20 features per finger (*M* = 80).

**Figure 5.**
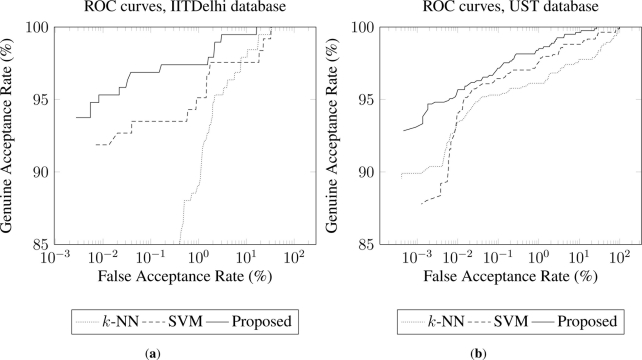
ROC curves for the proposed method in comparison to *k*-NN and SVM, using IITDelhi (**a**) and UST (**b**) databases. These results were obtained considering 4 samples for training, and 20 features per finger (*M* = 80).

Furthermore, performances of each method are provided by means of ROC (Receiver Operating Curve) curves [5,32], indicating the behaviour of the overall system. Concretely, Figure 4 presents the results of the three methods (proposed, *k*-NN and SVM) for the database GB2S. In addition, Figure 5 presents the results of the three methods (proposed, *k*-NN and SVM) for the databases IITDelhi [Figure 5(a)] and UST [Figure 5(b)].

Both Figures 4 and 5 illustrate that the proposed method improves the performance obtained by the other two methods along three contact-less databases.

### 5.3. Study of Performance Dependency on the Number of Training Samples

Biometric systems provide more precise results when more samples during the enrollment are acquired. The number of these samples coincides with the number of samples used to train the biometric system. Therefore, the study of the dependency between the performance of the whole system and the number of training samples is essential since an increment of the training samples will lead to an increment in performance, at expense of a diminution on the user acceptance and comfortability [11,12,35].

**Figure 6.**
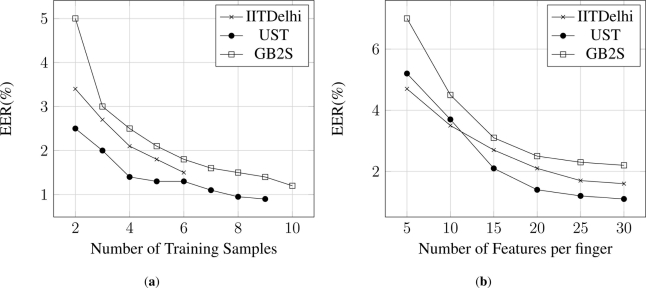
Comparative Equal Error Rate (EER) variation in relation to number of training samples to create the template and the number of features per finger, for the three databases: IITDelhi, UST and GB2S.

The performance of a biometric system is measured in terms of Equal Error Rate (EER) as defined in Section 5.1. The results are presented in Figure 6(**a**), where the variation of EER is presented along the number of training samples for each database. Due to the different number of samples per individual (7 for IITDelhi, 10 for UST and 20 for GB2S), the maximum number of training samples for IITDelhi is 6 and for UST is 9. In addition, Figure 6 was obtained fixing the number of extracted features to 20 per finger, *i.e.*, *M* = 80.

**Figure 7.**
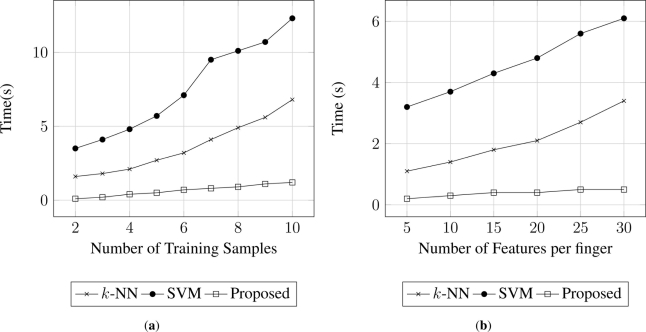
Comparative time variation in relation to number of training samples to create the template and the number of features per finger, for the proposed method, SVM and *k*-NN. Time is measured in seconds.

However, an increase in the number of training samples to create the template results in an increment of the time. Figure 7(**a**) provides the relation between time and number of training samples to extract the template. The proposed approach needs much less time to create the template since only considers samples from a single user, in contrast to SVM or *k*-NN where the template must consider samples from other users. Similarly, the values presented in Figure 7 were obtained fixing the number of extracted features to 20 per finger.

### 5.4. Study of Performance Dependency on the Number of Features

Together with the number of training samples, the number of features (distances) extracted from each hand is strongly related to the overall system performance. An increment on the number of features results in an increment of the performance, as well as in an increment of the computational cost.

Figure 6(**b**) contains the performance dependency on the number of features of the proposed method for the three databases: GB2S, IITDelhi and UST. This evaluation compares the evolution of the Equal Error Rate (EER) in relation to the number of features extracted for each hand.

In contrast, the computational cost increases substantially in relation to the number of features. More in detail, the computational cost contains both the time required to train the biometric system and the time needed to carry out the comparison. The latter time is negligible provided the computer specifications where experiments are carried out, since comparing a *M*-dimensional vector with the three approaches requires almost no time in comparison to other steps such as segmentation, feature extraction or the training of the biometric system.

In contrast, the number of features increases the processing time during the training. Figure 7(**b**) gathers the behaviour of the training time for the three systems (template-based, SVM and *k*-NN), in relation to the number of extracted features.

The results obtain in both Figures 6(**b**) and 7(**b**) were obtained fixing the number of training samples to 4, and considering only the GB2S database, assuming that similar results will be obtained for the other two databases.

### 5.5. Study of the Improvement Provided by the Feature Extraction Method

Apart from template creation, another innovative contribution of this paper consists of providing a feature extraction as described in Section 3.2.

Table 4 gathers the results obtained applying the proposed method and standard width feature extraction [2,3]. It shows that the use of this feature extraction method decreases the EER for each pattern recognition method, obtaining a remarkable improvement compared to standard extraction methods.

In addition, results presented in Table 4 where obtained by using the GB2S database. It is not difficult to assume that the feature extraction method conserves its properties, regardless the database.

Finally, the number of training samples was 4 and the number of feature extracted was also 20 per finger, as in all the evaluation scenarios.

## 6. Conclusions and Future Work

This paper has presented a biometric system based on hand geometry oriented to contact-less and platform-free scenarios. The contribution of this paper consisted of three innovative aspects: the proposal of a feature extraction method, invariant to distance to camera, hand rotation, hand pose and environmental conditions; the creation of a template involving only data (features) from one single individual; and a template matching able to minimize the intra-class similarity variation and maximize the inter-class likeliness.

The evaluation was carried out with three publicly available contact-less, platform-free databases, comparing the results obtained to two competitive pattern recognition techniques, namely Support Vector Machines (SVM) and *k*-Nearest Neighbour (*k*-NN), widely employed within the literature.

The results obtained show that the feature extraction method is able to provide invariant to changes features. In fact, the proposed method has achieved the second position in the Hand Geometric Points Detection International Competition HGC2011.

The template proposal only considers features from an individual. In other words, the template does not require information from the individuals contained on the rest of the database. This template creation not only reduces the computational cost of the enrollment procedure but also it allows biometric systems of one single individual, oriented to applications in mobile devices, for instance.

In fact, the use of both the feature extraction method and the template creation decreases remarkably the Equal Error Rate of the system, regardless the database involved. In addition, the feature extraction method improves the performance of the three compared approaches: the proposed method, SVM and *k*-NN. A further comparison to other existing feature extraction methods remains as future work.

Finally, the template matching proposed outcomes the presented pattern recognition techniques SVM and *k*-NN in terms of identification and verification performance. This template matching only considers those positions within the template with less intra-class variation, instead of comparing the whole template.

In general, the low computational cost required with this approach, together with the accurate performance in human identification makes of this proposed method a suitable scheme for devices with low hardware requirements, and its unconstrained and contact-less acquisition procedure can extend the applicability of this proposed system to a wide number of scenarios. In addition, there is no constraint on the quality of the camera during the acquisition, since one of the database was obtained with a mobile phone.

Considering future work, an implementation of this method in mobiles remains as future work together with its corresponding evaluation in real environments. Furthermore, more contact-less databases will be regarded for evaluation, together with the exploitation of both hands in a fusion scheme to improve identification and verification. Finally, an in depth evaluation of the effect of acquisition changes (distance-to-camera, hand rotation and openness variations) in identification performance will be considered.

## Figures and Tables

**Table 1.** Literature review on most recent works related to contact-less hand biometrics based on hand geometry. This table presents the relation between the features required for identification, the method proposed, the population involved together with the results obtained, in terms of Equal Error Rate (EER).

**Year**	**Ref.**	**Features**	**Method**	**Population Size**	**EER (%)**
2007	[5]	5–35 distances	Projective invariants	23	2.11
	[21]	23 distances	Entropy Discretization and SVM	100	5
	[4]	15 hand distances	SVM	18	8
	[27]	5 distances	AAM	18	5

2008	[18]	30–40 finger widths	SVM	20–30	4.2–6.3
	[26]	15 graph distances	DBNN	250	0.89
	[16]	Palmprint	Gabor Filters and SVM	49	1.7

2009	[7]	Zernike Descriptors	Fusion SVDD	86	1.5
	[14]	2D and 3D features	Savitzky-Golay filters	177	2.6
	[10]	Contour	DTW alignment	45	3.7
	[28]	40 distances	SVM	260	0.0035–5.7

2010	[15]	30 distances and angles	Correlation	50	4.2
	[25]	2D and 3D palmprint and geometry	Surface Code	114	0.71

**Table 2.** FTE and FTA rates for each database. These values will be considered during the calculation of FAR, FRR and EER rates in the evaluation.

	**GB2S**	**IITDelhi**	**UST**
FTE (%)	0	0.5	0
FTA (%)	0.4	0.7	0.2

**Table 3.** Equal Error Rate for each database and method. The results obtained with GB2S database are worst in comparison to the other databases since GB2S database present more variability in terms of hand rotation, distance to camera and environmental conditions. These results were obtained considering 4 samples for training, and 20 features per finger, *i.e.*, *M* = 80.

	**GB2S**	**IITDelhi**	**UST**
*k*-NN	4.3 ± 0.2	3.9 ± 0.2	3 ± 0.1
SVM	3.1 ± 0.1	2.4 ± 0.1	2.1 ± 0.2
Proposed	2.5 ± 0.2	2 ± 0.2	1.4 ± 0.1

**Table 4.** Comparative study of the improvement achieved by the proposed feature extraction method for each pattern recognition method (proposed, *k*-NN and SVM). The improvement achieved by the proposed method is remarkable. These results were obtained considering 4 samples for training, and 20 features per finger, *i.e.*, *M* = 80.

	**Standard Method [2,3]**	**Proposed Method**
*k*-NN	7.1 ± 0.2	4.3 ± 0.2
SVM	6.3 ± 0.2	3.1 ± 0.1
Proposed	4.8 ± 0.1	2.5 ± 0.2
